# Crowd-Based Cognitive Perception of the Physical World: Towards the Internet of Senses

**DOI:** 10.3390/s20092437

**Published:** 2020-04-25

**Authors:** Gianni Pasolini, Anna Guerra, Francesco Guidi, Nicolò Decarli, Davide Dardari

**Affiliations:** 1 WiLAB, CNIT, DEI, University of Bologna, Viale Risorgimento 2, 40136 Bologna, Italy; 2 WiLAB, CNIT, DEI, University of Bologna-Cesena Campus, Viale dell’Università 50, 47522 Cesena, Italy; anna.guerra3@unibo.it (A.G.); nicolo.decarli@unibo.it (N.D.); davide.dardari@unibo.it (D.D.); 3 WiLAB, CNIT, IEIIT, CNR, Viale Risorgimento 2, 40136 Bologna, Italy; francesco.guidi@ieiit.cnr.it

**Keywords:** Internet of Things, Cognitive Internet, Crowd Mapping, Crowd-Sensing, Personal Radar, Localization, Millimeter-waves

## Abstract

This paper introduces a possible architecture and discusses the research directions for the realization of the Cognitive Perceptual Internet (CPI), which is enabled by the convergence of wired and wireless communications, traditional sensor networks, mobile crowd-sensing, and machine learning techniques. The CPI concept stems from the fact that mobile devices, such as smartphones and wearables, are becoming an outstanding mean for zero-effort world-sensing and digitalization thanks to their pervasive diffusion and the increasing number of embedded sensors. Data collected by such devices provide unprecedented insights into the physical world that can be inferred through cognitive processes, thus originating a digital sixth sense. In this paper, we describe how the Internet can behave like a sensing brain, thus evolving into the Internet of Senses, with network-based cognitive perception and action capabilities built upon mobile crowd-sensing mechanisms. The new concept of hyper-map is envisioned as an efficient geo-referenced repository of knowledge about the physical world. Such knowledge is acquired and augmented through heterogeneous sensors, multi-user cooperation and distributed learning mechanisms. Furthermore, we indicate the possibility to accommodate proactive sensors, in addition to common reactive sensors such as cameras, antennas, thermometers and inertial measurement units, by exploiting massive antenna arrays at millimeter-waves to enhance mobile terminals perception capabilities as well as the range of new applications. Finally, we distillate some insights about the challenges arising in the realization of the CPI, corroborated by preliminary results, and we depict a futuristic scenario where the proposed Internet of Senses becomes true.

## 1. Introduction

One of the objectives of the Internet of Things (IoT) [1] is to map the physical world into the Internet, turning it into a *smart space* in which users can increase their knowledge of the environment and interact with it through their personal devices. In this respect, the ultimate goal is the mapping of the entire world, meant as the “Big Thing”.

This process started a couple of decades ago in outdoor scenarios, with the aim to derive digitized street maps for vehicular navigation systems. More recently, this mapping activity has been fueled also by volunteers through the OpenStreetMap project [2], that provides an openly licensed map of the entire world using free sources. Fostered by the success of such initiatives, the world mapping effort is still ongoing: in urban environments, highly descriptive maps are currently being obtained using cars equipped with sophisticated 2D/3D cameras, whereas indoor environments are the targets of large-scale initiatives, such as MapsIndoor [3] and Google Art Project [4], to allow visitors to easily navigate shopping malls, conference venues and museums.

Thanks to the pervasive diffusion of smartphones, maps so far generated using dedicated devices and infrastructures have been enriched through crowd-sourcing mechanisms, which allow people to share photos and other position-related information. However, visual and textual contents provide only a partial representation of the world, which is not informative about other position-related quantities that could be of interest, such as radio-frequency (RF) interference, sources of harvestable energy, geomagnetic field, temperature, humidity, or pollution.

The common way to explore this invisible side of the physical world is the deployment of wireless sensor networks (WSNs), whose introduction has stimulated a fertile research activity in the scientific and engineering community [5]. However, WSNs are generally characterized by significant deployment constraints, which do not make them the ultimate solution for the automatic and pervasive sensing of the physical world. Instead, the proliferation of mobile terminals equipped with a rich set of built-in sensors is creating a new class of sensor networks, denoted mobile crowd-sensing networks [6], which enable the collection of data with no dedicated sensing infrastructure or systems and take advantage of the dynamicity and pervasivity of sensing devices. This trend will be further fostered by beyond 5G networks, which are expected to integrate heterogeneous devices (smartphones, wearables, drones, IoT objects) with enriched sensing capabilities in a common joint communication and localization platform.

According to Ericsson [7], by 2030 there might be the advent of a new paradigm: the Internet of Senses (IoS). Giving credit to the utopian vision presented in [7], our thoughts will be fully accessible by the surrounding technologies thanks to the interface provided by our brains, so that we will be able to directly access the Internet space. From a more practical perspective, it makes sense to forecast that IoT devices will morph into the IoS, i.e., a pervasive technology that will allow humans to sense the world by means of a digital sixth sense, which complements the traditional five senses. The latter is the more pragmatic IoS vision elaborated by ZTE Corporation in the perspective of future 6G networks [8].

In this direction, the possibility to observe environment-related quantities thanks to the widespread diffusion of heterogeneous sensors, offers an unprecedented opportunity to augment the users’ perception and awareness of the environment. In fact, similarly to the five senses, whose outputs are processed by the brain to provide a richer experience of the surroundings and to “guide” the decision-making capability of humans, the synergies between built-in sensors of the same device or of different users’ devices will allow a cognitive supervisor to infer a deeper knowledge of the physical world. However, there is more to it. Differently from human senses, which are purely reactive as they passively sense some physical aspects of the world, electronic sensors can also be proactive, producing stimuli and observing the environment responses. In this regard, Nature gives us examples of sensors that proactively “interrogate” the environment with the purpose of learning and moving (e.g., bats’ sonars).

Whatever the nature of sensors, either proactive or reactive, the availability of a large and continuously updated knowledge-base (database of information) of the environment would increase the capabilities of humans and automatic systems to make complex decisions, for instance to navigate the environment or to optimize operations and processes.

Taking advantage of the affinities with cognitive neurosciences, the design of networks with cognitive capabilities, exploiting the synergies among reactive and proactive sensors, is expected to improve the level of environmental awareness (geo-awareness) that goes beyond the simple sum of gathered data [9]. The cognitive network of senses will be capable of inferring complex information from raw data, as well as focusing the attention on relevant elements, driving the sensing process. For instance, it will be possible to properly control or to adapt the interrogation of proactive sensors based on the acquired experience.

Finally, while most of what perceived by a person typically remains a private experience, or is only partially shared with a small group of people, the networking capabilities provided by the Internet and the power of crowds to perform pervasive sensing tasks, open unexplored opportunities to tie the virtual and the physical worlds, in which humans are enclosed in the loop both as (raw and partial) data providers and recipients of “cooked” information.

With this vision in mind, in this paper we introduce the Cognitive Perceptual Internet (CPI), which encompasses devices, infrastructures, and algorithms (the global Internet, sensors networks, crowd-sensing applications, learning and cognitive processes), and integrates them into a pervasive, comprehensive, cognitive network with both reactive and proactive sensing, able to properly map the physical world into the virtual world. The IoS, meant as the digital sixth sense, is thus the enriched vision of the world provided to users by the underlying CPI.

The purpose of this paper is twofold: (i) to introduce a brain-inspired architecture of the CPI, also providing some preliminary results, and ii) to discuss the open research fields and to identify the major issues to be addressed to turn the CPI into reality. In the remainder of the paper we describe the principles of the CPI, with the intention to discuss its peculiarities and to spark new interests and developments in this field. Our CPI vision is also exemplified by a couple of experimental demonstrations, which, although incomplete and limited in their extent, provide some glimpses of the much wider CPI picture.

The paper is organized as follows. The related works are outlined in Section 2 and the CPI is proposed in Section 3. Its architecture is introduced in Section 4, whereas important research challenges relevant to its realization are discussed in Section 5 and Section 6, which concern cooperation schemes and infrastructureless localization and mapping. Section 7 describes preliminary experimental demonstrations in the direction of the CPI, whereas the conclusions are drawn in Section 8.

## 2. Related Work

The IoS paradigm has recently gained momentum as a target for the future 6G technology, being thus investigated by prominent telecommunications manufacturers such as Ericsson and ZTE Corporation [7,8]. Clearly, an IoS-enabled scenario requires a big advancement of the global communication network, which is expected to handle a significant traffic increase and to provide a massive data capacity. Most importantly, it must feature perceptual and cognitive capabilities, such as learning, attention and inference, to effectively complement human senses.

Our paper addresses some of the challenges posed by the IoS, and is motivated by the absence in the scientific literature of a comprehensive discussion on the technologies and methods supporting this paradigm. In fact, the different topics at the basis of the IoS, spanning from crowd-sensing and multi-user cooperation to distributed learning and cognitive mechanisms, have been so far investigated only individually in their specificity. For the sake of conciseness, in the following we focus our literature survey on mobile crowd-sensing, which is the foundation on which the whole IoS is built upon. Indeed, what is missing is a unique framework where mobile crowd-sensing is empowered with high level functionalities (namely perception, learning, inference and cognition), as in the proposed CPI paradigm.

*Mobile crowd-sensing.* As anticipated, the direct involvement of people in the sensing loop is one of the peculiar features of the proposed system. In this regard, mobile crowd-sensing [10,11] is expected to move a step further, with user participation being both implicit or explicit, and mobile social networks and sensing acting as data sources [12]. By taking advantage of the power of the crowd, a plethora of different applications has already been enhanced [13,14,15,16,17,18,19,20,21,22,23,24,25]. As an example, in [13], the crowd-sensing problem is solved by acquiring real-time data using 5G technologies, and by extracting features from the captured data adopting a novel deep learning approach. The users’ willing and privacy are also considered by developing an encryption procedure for safety protection.

To foster global cooperation among users, a novel approach, called *social incentive mechanism*, has been proposed in [16], which leverages the social relationships among participants. The large amount of available data has also been exploited to detect malicious users [17], or by envisioning the potential of deep-fused human-machine systems [18]. A solution tailored for healthcare has been considered in [19], by proposing an architecture to connect intelligent things in smart hospitals based on narrowband-IoT, with the introduction of edge computing to deal with the requirement of latency in medical process.

Crowd-sensing has been used also for monitoring several environmental parameters [20,21,25], or for traffic prediction [14,22]. Recently, the concept of sparse mobile crowd-sensing has been introduced, which exploits the spatial and temporal correlation among data sensed in different sub-areas to reduce the number of sensing tasks. In this way, the overall sensing cost in terms of smartphone energy consumption and incentives can be reduced, while guaranteeing high performance [23].

When considered in the IoS perspective, the above mentioned papers (and references therein on the same topic, not cited here for the sake of conciseness) suffer from the same shortcoming: each of them investigates a specific aspect, so that none of them provides a general framework of a global network that relies on distributed cognitive capabilities to coordinate its operations, from the data exchange policies to the information inference.

*Systems with cognitive capabilities*. As a matter of fact, cognitive capabilities have been already introduced in electronic sensing devices, such as radars. In this case, the objective was to make them learn from the environment and to behave according to the acquired experience [26,27]. In particular, cognition was referred to as the capability of a radar of continuously learning about the environment through interactions, then driving the receiver and the transmitter actions according to the already gained experience. Research in this direction is extremely vibrant, as proven by many papers published in recent years [28,29,30,31,32,33] that have paved the way to future trends of investigation, often intertwined with the domain of artificial intelligence.

*The roots of the CPI architecture*. To face the challenges posed by the IoS paradigm, a similar step needs to be taken also in the field of mobile crowd-sensing. This is where our CPI architecture plays a major role. As will be clear in Section 4, the architecture we propose, which gives the network its cognitive capabilities, is inspired by the model of the human *working memory* proposed by Baddeley in [34]. Despite its origin in the neuroscience field, the *working memory* can also be regarded as a cognitive dynamic system, whose concept was introduced by Haykin and Fuster in [35]. Therefore, our CPI architecture has its roots in this relatively recent discipline, which weaves concepts belonging to the ICT field, such as machine learning, statistical signal processing, stochastic control, sensor fusion and information theory, and ideas drawn from neuroscience, statistical learning theory, and game theory.

*System of systems approach for the CPI*. Clearly, the potential complexity of such an architecture, which includes myriads of devices and network elements, is a major challenge to be addressed. How can the numerous “network pieces” be integrated into a usable whole? This kind of problem is usually faced adopting a *system of systems* approach, which has been studied since decades [36,37,38]. With reference to scenarios somehow related to this paper, it was recently adopted to investigate crowd mobility in smart cities by means of IoT technologies [39].

In this paper, the proposed CPI architecture is designed according to the same *system of systems* approach, as it will be clarified in Section 4.

## 3. The Cognitive Perceptual Internet

As summarized in the previous section, a rich literature exists regarding crowd-sensing/sourcing applied to different contexts. However, most works address only specific problems (e.g., privacy, user incentive, energy efficiency, etc.) or propose solutions that are tailored to specific applications using reactive sensors. Proactive sensors (e.g., radars) with cognitive capabilities have been considered only with reference to single systems [27]. Therefore, there is a lack of a general vision of a global network capable of sensing and mapping the physical world using both reactive and proactive sensors and exploiting cognition capabilities, as done by human senses and brain.

In view of the above considerations, the CPI concept introduced in this paper is aimed at broadening the scope and the functionalities of the global Internet, turning it into a cognitive system with active and passive perception capabilities based on mobile crowd-sensing mechanisms as well as traditional sensor networks. The CPI is thus conceived as a worldwide “system of systems”, which encompasses infrastructures and devices, such as the global Internet, all national cellular networks and a large amount of communication-enabled sensing devices (including smartphones, wearables, and “smart objects” in general), as well as immaterial components (processing algorithms and stored data) which enable complex cognitive capabilities (e.g., learning, inference, pattern recognition, stereopsis, scene assembly, analysis of spatial relations, object search and semantic processing).

**Figure 1:**
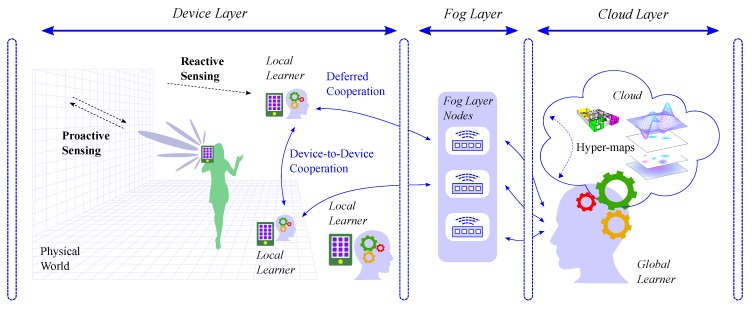
CPI functional scheme.

Our functional vision of the CPI is sketched in Figure 1, where several mobile nodes (e.g., 5G smartphones) equipped with heterogeneous sensors (e.g., reactive and proactive, vision- and radio-based), acquire environment-related quantities. Such devices, denoted as “local learners” in Figure 1, collect data that represent a partial, spatially limited, description (that is, a chunk) of some physical characteristic of the surroundings. In this scenario, the CPI addresses the way such data are collected (with reactive or proactive sensors), processed (locally or remotely), exchanged (among neighbors or through the Cloud, in one direction or both), analyzed (by cognitively enabled applications or traditional ones) and stored (how and where). In Figure 1, the CPI is illustrated by highlighting some of its elements, that are: (i) the reactive and proactive sensing capabilities of mobile terminals, (ii) the bidirectional communication links connecting neighboring local learners together and all local learners with the “global learner” located in the Cloud, and iii) the cognitive capabilities, symbolized by the “thinking gears”, possessed by all of them, although with some differences.

Thanks to the CPI, the crowd-sensing concept is generalized, being no more a mere and agnostic zero-effort solution to automatically collect spatially distributed measurements. Rather, it is intended as a component of a distributed learning network, with both reactive and proactive sensors, providing sophisticated cognitive functions such as perception, attention, memory and inference similarly to what happens in living organisms. Within the CPI framework, the Internet can be seen as a network that “learns” about the physical world through interactions among distributed heterogeneous sensors, exploiting mobile crowd-sensing mechanism, thus supporting users’ actions with context-aware information.

This vision leads also to the *hyper-map* concept, which is meant as an efficient geo-referenced repository of knowledge about the physical world acquired through sensing, multi-user cooperation, distributed learning and semantic processing of data. It consists of a set of maps stored in the Cloud, describing some properties of the environment derived from data sensed by the crowd in different locations. However, maps are not mere distinct and punctual representations (point estimates) of the quantities of interest. The whole hyper-map is conceived, instead, as a unitary structured record of sensed information (represented by numerical data) and inferred knowledge (their meanings and cross-relations) gained through cognitive methods. It may incorporate, for instance, statistical information on the confidence level of sensed data as well as the results of elaborated processing aimed at abstracting a general feature.

The information gain is obtained by exploiting both direct user cooperation, through device-to-device (D2D) communications (as envisioned in 5G and beyond networks), and deferred cooperation mediated by the network (Cloud). By cooperating, users enlarge their domain of exploration, enrich their ability to learn more, and refine their local view of the environment.

Indeed, the scenario envisioned in this paper is very far-looking and several issues must be addressed to turn the CPI into reality, mainly concerning the overall architecture, the enabling technologies, the service requirements and the data-processing. As far as we can discern, the most relevant ones are related to:

1. *Overall architecture of the CPI*, capable of efficiently exchanging, extracting and organizing the relevant information from raw data, as well as of inferring complex information from them. This aspect is discussed in Section 4;
2. *Cooperation model*, to coordinate the information exchange among users to minimize the communication burden. Depending on the situation, it can be direct, through D2D communications, or Cloud-based (deferred). This aspect is discussed in Section 5;
3. *Infrastructureless localization and mapping*, which is required to geolocalize sensed data where no positioning infrastructure is available. This aspect is discussed in Section 6;
4. *Efficient representation of the knowledge acquired*, which should be the lightweight distillation of the more significant data generated by the crowd (whose amount could be huge). A possible solution is proposed in Section 7.1;
5. *Sensing devices*, which need to be integrated in small-size, energy limited, personal devices. The integration of proactive sensors is, in particular, a challenging task. An example of proactive sensor that could be conveniently embedded in future smartphones is provided in Section 7.2.

In addition to the above listed issues, there are other aspects, not discussed in the following that need to be addressed. Among these, *privacy* is one of the most important. In fact, collected data are usually tagged with important contextual information, such as sensing location and time. Clearly, the disclosure of such data can have serious implications on users’ privacy. Moreover, multiple reports from the same user can be linked to infer even more private information, such as home or office addresses. In this regard, proper countermeasures should be implemented [40,41].

## 4. Architecture of the Cognitive Perceptual Internet

As previously stated, there has been a recent attempt to bridge the gap between cognitive neuroscience and electronic sensing by exploiting the analogies with human perception and decision-making. The aim was the introduction of cognitively controlled sensing into tracking radar systems [9]. However, the path toward the adoption of cognitive neuroscience models into artificial sensing is still long, especially in the crowd-sensing perspective. In fact, the focus of cognitive neuroscience is on single brain cognition and not on the global cognitive perception of a group of people interacting with one another [42]. Nonetheless, the model of the human *working memory* proposed by Baddeley [34] provides an inspiring example for the CPI architecture, as it will described in the following.

### 4.1. The Human Working Memory Model

**Figure 2:**
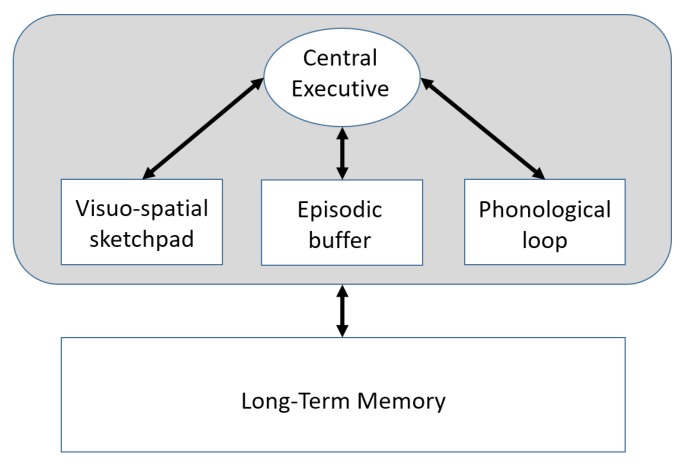
Human brain working memory model [34].

In the neuroscience field, the concept of working memory denotes a system that provides an interface between perception, action and long-term memory. It incorporates storage and processing, as both functions must be concurrently engaged to enable the cognitive aptitude exhibited by human brains. As depicted in Figure 2, the Baddeley’s working memory model is a multi-component system. The phonological loop and the visuo-spatial sketchpad are short-term, limited capacity, memories, which act as interfaces between audio/visual sensing and action/cognition. The former temporarily stores a limited amount of phonological information (words, letters and digits) perceived by the sense of hearing. It is used, for instance, to remember a phone number in the short-term. The latter temporarily stores visual and spatial information perceived by the sense of sight. It can be used, for example, for constructing and manipulating visual images and for representing mental maps.

Short-term audio and visual-spatial information, temporarily stored in the aforementioned buffers, are possibly linked one to another in the third component, namely the episodic buffer, which is a limited capacity memory that is capable of binding together information from several different sources into unitary multimodal chunks or episodes (hence the term “episodic”). Its presence explains short-term memory of features that do not match the audio and visuo-spatial stores as well as the evidence of cross-domain associations in working memory, such as the retention of links between names and faces.

Verbal-phonological and visuo-spatial representations are managed and manipulated with the help of attention-related processes controlled by the *Central Executive*, which is the attentional mechanism of the working memory. The Central Executive plays the role of control center, being responsible for directing attention to relevant information, inhibiting signals or responses, supervising the integration of information and coordinating cognitive processes when more than one task is simultaneously performed. It is also supposed to manage the access to the long-term memory, to retrieve or store information.

### 4.2. The Brain-Inspired CPI Architecture

**Figure 3:**
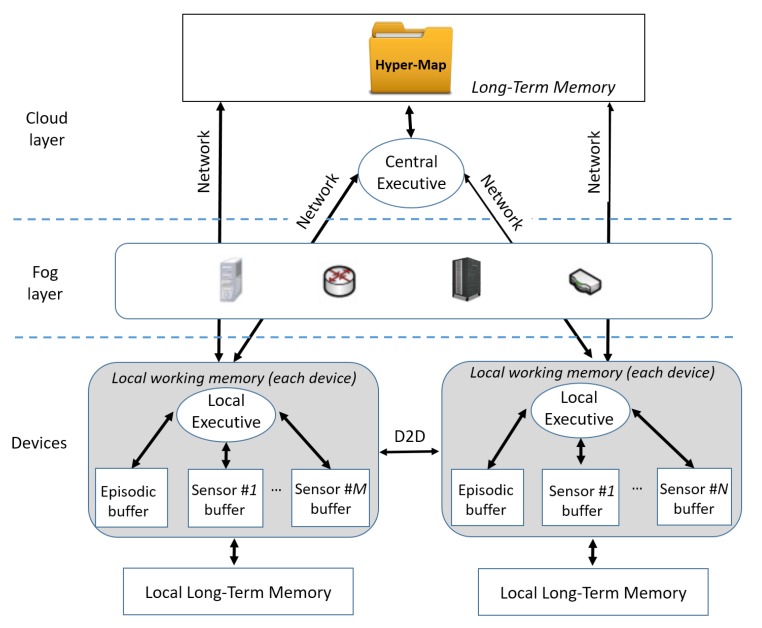
The CPI architecture.

Inspired by the “single brain” working memory envisioned by Baddeley, a similar, yet extended, architecture is here proposed for the “multi-brain” CPI, which is depicted in Figure 3. Since a multitude of mobile terminals with sensing and processing capabilities is involved in the cognitive tasks of the CPI, there are as many working memories as there are devices, each of which is a *local learner* (see also Figure 1).

**Short-term buffers in the CPI.** The CPI-correspondents of the human phonological loop and visuo-spatial sketchpad are the on-board local buffers, where the outputs of local sensors are temporarily stored. Such raw data (e.g., camera pictures or proactive sensing outcomes) might be somehow related one to another through a possible in-device “data fusion” process, capable of finding meaningful associations, and stored in the local episodic buffer, which temporarily holds an enriched, although spatially limited, description of the surroundings.

**Local executive in the CPI.** The Local Executive (*local learner*) is in charge of managing local cognitive operations (sensing, storage and processing), focusing the attention on relevant information, inhibiting useless sensing, supervising data integration, also taking into account more general aspects such as the energy consumption and the computational burden. To retrieve missing data, it might use the communication network to access the hyper-map (that is, the long-term memory) located somewhere in the Cloud. Moreover, depending on the characteristics of local information (e.g., small-scale/global relevance in time/space), the Local Executive might also trigger its transmission towards nearby devices through D2D communications or towards the Cloud, to update the general hyper-map.

**Central Executive in CPI.** Moving to the highest hierarchical level, the Central Executive is the global supervisor (*global learner*), which plays the role of “brain of brains”. It interacts with users’ devices, forcing or inhibiting (e.g., for privacy reasons) the sensing. Moreover, it performs heavy data-processing tasks and oversees all operations involving the hyper-map. In particular, it can distill the relevant information content, finding associations between large-scale, possibly heterogeneous data, and controlling the information flow.

**Long-term memory in CPI.** Finally, the hyper-map deputizes the human long-term memory, since multi-domain information (e.g., visual data, RF interference, pollution, harvestable energy) are stored according to semantic criteria (i.e., based on relevance, meaning and association). Hyper-maps shall capture only the significant geo-referenced information contained in the observed phenomena, considering that they could involve heterogeneous, correlated, and time-varying quantities with both random and quasi-periodic behavior. A simple example of hyper-map representation and construction is provided in Section 7.

From the implementation point of view, the recently introduced deep neural networks represent powerful tools to realize both local and global-learners, as well as short- and long-term memories [43]. In this regard, an open research topic is how to let distributed deep neural networks cooperate/interact each other to accomplish the learning and hyper-map tasks.

Finally, it is worth stressing that both the Central Executive and the hyper-map should be intended as logical entities, whose implementations do not need to be centralized in a strict sense. In fact, as highlighted in Figure 3, the principles of distributed computing and distributed database systems could be conveniently applied for their implementation, according to the classic Cloud paradigms [44,45]. Moreover, possible challenges and constraints in network connectivity, communication bandwidth and service latency can be successfully addressed by Edge and Fog computing infrastructures, in which data, computing capabilities, storage and applications are located somewhere between the sensing devices and the Cloud.

### 4.3. Relationship with Cognitive Dynamic Systems

Our conceptual imitation of the human working memory for the CPI architecture belongs to the wide topic of *cognitive dynamic systems*, which is addressed by the inspiring paper authored by Haykin and Fuster [35]. This discipline builds on ideas in machine learning, statistical signal processing, stochastic control, sensor fusion and information theory, and weaves them into new ones drawn from neuroscience, statistical learning theory, and game theory. According to [46], cognitive dynamic systems *“will provide principled tools for the design and development of a new generation of wireless dynamic systems”*.

In particular, the *working memory model* depicted in Figure 2 can be easily mapped into the block diagram of the *basic cognitive dynamic system* proposed in Figure 1 of [47]. More specifically, the Central Executive block shown in our Figure 2 corresponds to the Controller of Figure 1→[47]. In fact, both play the role of control center, being responsible for directing attention to relevant information, inhibiting signals or responses, supervising the integration of information and coordinating cognitive processes. Further exploring the parallel between our Figure 2 and the block diagram presented in Figure 1→[47], the *visuo-spatial sketchpad*, *episodic buffer* and *phonological loop* represent the short-term memory shown in Figure 1→[47]. As for the long-term memory of Figure 2, it corresponds to the executive memory of the dynamic system block diagram, as, according to [47], *“long-term experiences acquired from the past (...) have been stored in the executive memory”*.

We want to emphasize that the straightforward parallel between the *working memory model* of our Figure 2 and the block diagram of dynamic systems taken from [47] shows the link between cognitive neuroscience and artificial cognitive systems, translating neuroscience concepts and terminology into engineering systems and tools.

### 4.4. Working Memory and the CPI: Concluding Remarks

Clearly, the brain-inspired model depicted in Figure 3 is only one of the possible CPI architectures. Other solutions might prove to be better suited to the envisioned IoS scenario. In fact, much work must be done to design and implement the data-processing and decision-making strategies at local and central levels. This is certainly a difficult task that entails a resource management optimization which should balance different aspects, such as energy consumption, computational effort, local/general relevance, and timeliness. New research threads will emerge from the CPI, which are expected to spark new developments in many directions.

## 5. Cooperation Schemes in Crowd-Sensing Networks

Differently from cognitive neuroscience models, which consider a single brain and five senses, the possibility to have a huge number of mobile users cooperating through direct and deferred communications opens the new fascinating perspective of extending the study from a cognitive perceptual brain to a network of cognitive perceptual brains, as envisioned by the CPI. Making a parallel with human experience, direct cooperation corresponds to information exchanged between two people. In many cases, such information has only a local or short-term relevance and hence it is useless to spread it towards distant users. In such cases, direct cooperation via D2D communications is preferable. On the contrary, deferred cooperation might correspond to information exchange through books in a library, which is a repository of large-scale/long-term information. In the CPI vision, deferred communications take place through the hyper-map in the Cloud, which plays the role of the library.

Network-based knowledge-inferring schemes, such as those based on Bayesian learners [35], through the adoption of multi-scale state-space models, are expected to be important tools to capture this local/short-term and large-scale/long-term dichotomy. Other tools are given by deep Gaussian processes, or deep learning schemes in general, which allow several data abstraction layers [48].

In this context, the communication strategy should pursue the optimal balance between local and global knowledge/learning in terms of energy efficiency, communication cost and hyper-map representation quality. Multi-scale state-space models could be adopted both by the Central Executive and the Local Executives to efficiently manage the knowledge acquired by sensors, as they allow discrimination between local and global information and facilitate the design of ad-hoc network-based statistical inferring schemes. For example, such models are expected to be well-suited to characterize physical phenomena experiencing cyclostationarity at different time scales (e.g., hours, days, months, seasons) or space scales (e.g., person, room, building, city, etc.), thus allowing to separate the information to be exchanged only directly between close users (through D2D communications), from that involving the access to the hyper-map. However, specific investigations are required to assess limits and potentials of such models in the CPI scenarios, as well as those of new models specifically conceived to fit the CPI peculiarities.

Special attention deserves the way users should contribute to the sensing process: on the one hand, users could be almost non-participating, being only required, for instance, to switch on their devices’ sensors. On the other hand, they could be fully participatory, for instance moving around according to the sensing-task needs and the Central Executive feedback received through the network. In all cases, proper incentives should be provided to motivate users to share their own resources (e.g., battery and data traffic) for sensing tasks. This is a well investigated issue, and many strategies, especially designed for mobile crowd-sensing scenarios, have been proposed, which mainly fall into three categories: entertainment, service exchange, and money. A comprehensive survey on this topic is reported in [49].

Finally, approaches to exploit the neuroscience concept of *attention* is another interesting open point to investigate in order to improve opportunistic cooperations between nodes. In fact, as in humans the function of attention is aimed at an efficient allocation and management of resources in the brain [42], one sensor might avoid to sense a certain area, thus saving energy and radio resources, if a sufficiently accurate knowledge of that area has been already acquired by other users.

## 6. Infrastructureless Localization and Mapping

Generating the hyper-map requires the knowledge of the spatial coordinates of data sensed by the crowd. In outdoor scenarios, such information can be acquired thanks to satellite positioning systems. Contrarily, ad-hoc solutions are needed in indoor scenarios where the GNSS is not always available or reliable.

Localization techniques for indoor environments have been deeply studied in the last 15 years and several commercial systems are available. The most promising solution in terms of accuracy/cost tradeoff is given by the ultrawide-band (UWB) technology. Unfortunately, like most solutions, it needs the deployment of dedicated infrastructures that prevents its adoption in every situation [50]. Moreover, even in the presence of a dedicated infrastructure, a highly accurate localization within indoor spaces needs the knowledge of the environment map, whose derivation requires, in turn, an accurate localization technology.

This “chicken or the egg” dilemma has been addressed by the signal processing community by introducing simultaneous localization and mapping (SLAM) methods taking advantage of the high capability of LiDAR or stereoscopic cameras (Visual SLAM) to discriminate scene features [50]. In traditional SLAM, a laser-equipped or camera-equipped agent explores an unknown indoor environment and estimates the corresponding map while simultaneously keeping track of its location within it. The shortcomings of traditional SLAM approaches are related to the need for perfect visibility conditions and, above all, to the mechanical steering of the scanners. When moving towards a large-scale diffusion perspective, as in crowd-sensing scenarios, these systems do not represent feasible solutions to be embedded in personal devices, with limited energy/cost/dimension, and requires the active participation of users that should, at least, properly manage the device.

To overcome these shortcomings, SLAM-like techniques might be extended to include reconfigurable massive antenna arrays working at millimeter waves (mmW) with electronic steering capabilities [51]. In fact, apart from using these arrays to improve the communication quality, they can be good candidates to enable high-definition radar-like applications, namely *personal radars*, which exploit the beamsteering capability to automatically scan and proactively interrogate the environment with mmW probe signals, thus obtaining 3D mapping and self-localization functionalities with no need for active user participation. Differently from stereoscopic cameras and LiDARs, the use of RF signals has the advantage of being cheap, energy efficient and “technologically dense”, as communication and localization tasks would rely on the same hardware.

It is worth stressing that as with all SLAM devices, personal radars not only provide localization information, which is the focus of this section, but also the raw data to extract the local map of the environment. This aspect will be discussed in Section 7.2.

## 7. Numerical Results: Examples of Crowd Mapping and Proactive Sensors

In the following, we will provide two examples of experimental results concerning two building blocks of the CPI architecture.

### 7.1. Crowd Mapping with Reactive Sensors

In this section, we illustrate an example of crowd mapping of a spatial field using randomly moving wireless sensors. Specifically, we consider a scenario where measurements of a certain spatial field are taken in random known positions and, then, processed by the Central Executive to infer the overall distribution of such a spatial field in the whole region of interest, including positions where measurements have not been collected. This task poses severe challenges, which are mainly related to the fact that data are taken in random locations [52] and that they are affected by noise. Moreover, the amount of data collected might increase with time to an extent that their storage and processing may become unfeasible. This calls for efficient statistical representations of spatial fields as well as for processing algorithms whose computational complexity is eventually independent of the number of measurements.

In this regard, a powerful tool is represented by the theory of Gaussian processes (GPs), which is widely adopted in machine learning [53,54]. However, its adoption in the context of crowd mapping for spatial field estimations might still suffer from complexity and memory issues.

**Figure 4:**
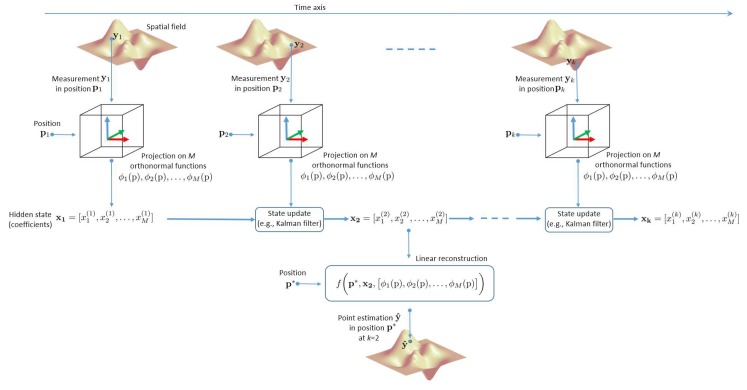
Hidden Markov process applied to crowd mapping.

To tackle this issue, a combined GP state-space method, whose complexity and memory requirements do not depend on the number of measured data, is proposed in [55]. The main idea is sketched in Figure 4: in a given region of interest, where each point is characterized by spatial coordinates p, the spatial field to be estimated is modeled as a GP expressed as a linear combination, with Gaussian random coefficients, of a finite number *M* of orthonormal basis functions $\phi_{1}\left(p\right),\phi_{2}\left(p\right),\ldots ,\phi_{M}\left(p\right)$ (e.g., the 2D Fourier series). In particular, such coefficients are obtained projecting each new measurement $y_{k}$, taken in position $p_{k}$ at the discrete time instant *k*, onto the orthonormal basis, as shown in Figure 4. These coefficients, collected in the vector $x_{k}$, can be regarded as the state, at the *k*-th observation instant, of the GP state-space model described by a hidden Markov process.

The number *M* of coefficients considered (which equals the number of orthonormal basis functions) is fixed and depends on the spatial variability of the physical field (spatial bandwidth). When a new noisy measurement $y_{k+1}$ is taken at position $p_{k+1}$, it is projected onto the orthonormal basis and used to update the state from $x_{k}$ to $x_{k+1}$, whose length does not change.

Under the hypothesis of Gaussian measurement noise, since all random variables (RVs) are Gaussian and the transformation from the state vector to the spatial field is linear, the statistical evolution of the state can be efficiently computed through a Kalman filter algorithm, which computes the new vector of coefficients $x_{k+1}$ starting from both $x_{k}$ and the projection of the new measurement, with a complexity independent of the number of measurements collected so far (see Figure 4).

When needed, one can compute a point estimate of the GP in a certain position $p^{*}$ conditioned to the history of measurements $\left[y_{1},y_{2},\cdots ,y_{k}\right]$ by applying the linear combination of the orthonormal basis with the coefficients in the current state $x_{k}$, which is the knowledge about the GP acquired up to the discrete time instant *k*. In this way, it is not necessary to keep in memory all measurements, whose number could explode. Specifically, the amount of memory and the computational burden are proportional to *M* (that is, to the number of functions of the orthonormal basis) and not to the increasing number *N* of collected measurements.

It is worth observing that the Kalman algorithm provides, as a side result, also the covariance matrix of the state $x_{k}$, which is a measure of the state uncertainty. The complete statistical description of $x_{k}$ (conditional mean and covariance matrix) can thus be stored in the hyper-map, which is, at the same time, very compact and highly informative, as it also provides the information uncertainty.

**Figure 5:**
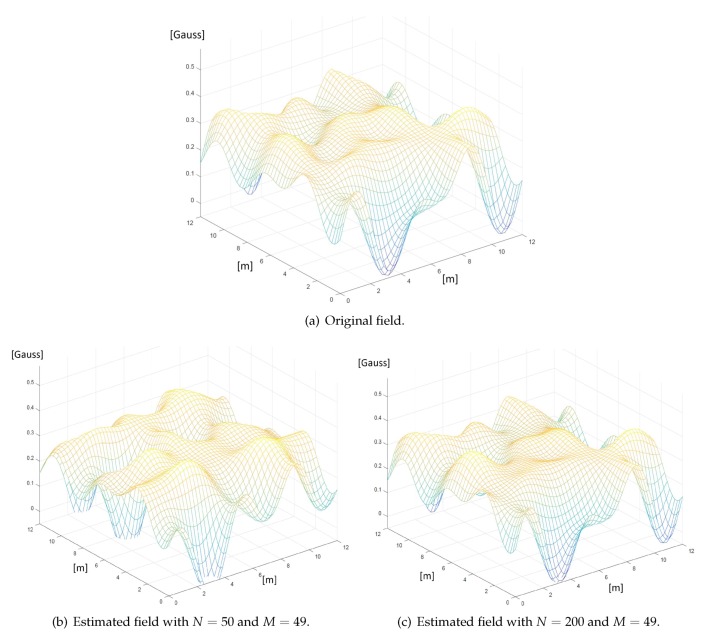
Original (**a**) and estimated magnetic fields (**b**,**c**).

Figure 5 shows an example of experimental validation of the GP-state-space method applied to the mapping of the geomagnetic field through mobile magnetic sensors. During the experimental campaign, the modulus of the actual geomagnetic field was measured in a geo-referenced dense grid of G=4765 points within the 11 × 11 m${}^{2}$ monitored area. This rich set of data has provided a spatial function very close to the original field, shown in Figure 5a, used as reference to assess the performance of the GP-state-space method. Then, the presence of mobile users traveling along random paths and collecting a total amount N≪G of magnetic field measurements was emulated. Figure 5b,c show the estimated (i.e., reconstructed) fields when N=50 and N=200 measurements have been taken, respectively, assuming perfect knowledge of the measurement positions. One observes that the reconstruction accuracy increases with the number of measurements and it is quite good with N=200 measurements. More details can be found in [55,56,57].

In conclusion, the illustrated method allows an efficient statistical characterization of the investigated spatial field, which can be easily updated once new data become available. This is especially appealing for distributed sensing applications as in the CPI, where a huge amount of data is expected to be collected in random locations. In particular, this might be a possible solution to efficiently store in the hyper-map information on spatial fields. However, depending on the specific nature of collected data, alternative strategies could be adopted. For instance, GPs are not the proper representation for information that cannot be represented as spatial fields or for information with a sparse spatial character. How to efficiently describe such information, possibly with their uncertainty and considering their possible time variability, is definitely an open research field.

### 7.2. Mapping with Proactive Sensors

The introduction in the CPI architecture of proactive sensors and their integration with reactive ones is a new field of investigation rich of opportunities to enhance the information content of the hyper-map, but it also generates new unaddressed issues. On the one hand, most of distributed sensing studies have only considered reactive sensors. On the other hand, proactive sensing has been investigated in single-user scenarios [9], so that there is a lack of studies in cooperative/social and crowd scenarios.

**Figure 6:**
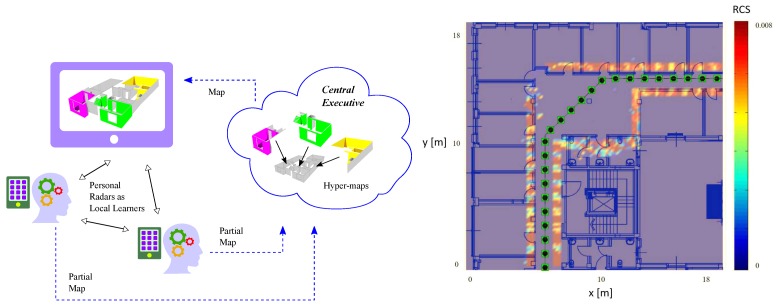
Example of next generation smartphone-centric service: the personal radar (**left**); example of mapping result in a real indoor environment using a personal radar (**right**).

Recently, the personal radar concept has been proposed as a smartphone-centric low cost solution for indoor mapping and navigation (Figure 6(left)) [58], which exploits mmW arrays that will be likely introduced in 5G and beyond smartphones. In fact, the reduced wavelength at mmW allows for packing hundreds of antennas into a credit card-sized area, providing future smartphones with pencil-shaped steering beams. In addition to providing high antenna directivity during ordinary communication activities, such a feature will also allow the accurate 3D scanning of the environment through the electronic-steerable transmission of RF signals and the collection of radio echoes reflected by walls and objects [58]. This will turn the RF front-end of smartphones in powerful proactive sensors.

The crowd will be involved in this process since different users, freely moving within the space to be mapped, might provide partial views of the surroundings that will be combined at the Central Executive to generate highly accurate 3D models of the whole environment (see Figure 6(left)) and, in principle, of the entire indoor world, thus originating the crowd radar concept. Then, such information will be shared with other (contemporary or future) users for possible mapping refinements as well as for the provision of navigation services. Moreover, images captured using cameras could be included in the acquisition process to create texturized 3D visual maps.

In this direction, a case study adopting a new grid-based Bayesian state-space model, which properly accounts in the observation model the rich set of measurements and the radar cross section (RCS) of the objects (including walls) populating the environment, has been proposed in [58]. The state-space approach has been followed to represent and update the acquired knowledge about the surrounding map, in a similar way as summarized in Section 7.1; in this case, the state vector represents the RCSs of all cells (i.e., elementary square regions of a grid) into which the environment is discretized.

An example of the feasibility of the personal radar concept and of indoor map reconstruction at 60 GHz is reported in Figure 6(right) [58], where the estimated 2D map obtained from an antenna array of 225 elements is reported. The black-filled dots represent the path followed by the mobile user. Examples of experimental results obtained using a massive antenna array can be found in [59].

We underline that observations are affected by positioning errors and wireless channel impairments (e.g., multipath fading) that make the derivation of accurate maps a major challenge. The contribution of many users (crowd-sensing) becomes essential to cope with such issues. In fact, the availability of many radar images, generated by different devices with different perspectives, is expected to refine the maps and, hence, increase their accuracy. This requires, however, ad-hoc algorithms that are capable of properly mixing different radar images.

More in general, research activities in the personal radar field, which represents a fundamental pillar of the CPI architecture, are at the initial steps, and further developments are required in several directions, which include, for instance, accurate beamshaping and beamsteering, radar images fusion, map extraction from radar images, and map representation with confidence level information.

### 7.3. Final Remarks on the Examples

The experimental demonstrations described in Section 7.1 and Section 7.2, although incomplete and limited in their extent, provide an insight on the huge potential impact of the CPI architecture. In the former example we showed how to distill and efficiently represent the knowledge acquired thanks to the measurements collected by the crowd, whereas in the latter we considered a single user adopting a proactive sensor (the *personal radar*) to effortlessly derive maps of indoors.

The CPI would allow “closing of the loop”, making it possible to share and integrate the partial maps derived by each single user, thus exploiting the power of the crowd, and stimulate the proactive sensing of missing data as well as to efficiently store and disseminate the acquired knowledge, taking advantage of the intelligent strategy adopted to represent the information gathered in the hyper-map. Ultimately, the CPI would provide the tools to cognitively supervise and tightly intertwine different aspects of the world-sensing process that are currently separately considered, significantly enhancing the overall outcome and offering a system-oriented vision of the problem.

## 8. Conclusions and Future Perspectives

The CPI architecture introduced in this paper is expected to create a fertile setting for new ground-breaking applications. For instance, crowd radar networks, exploiting mmW massive arrays in future smartphones, will stimulate the diffusion of user-generated high-definition 3D information of indoors. We envision a future where highly accurate 3D indoor maps of potentially all buildings in the world will be easily created and made freely available as open data (according to their privacy level) for the development of new CPI-enabled technologies supporting, for instance, over aging or vision impaired persons.

More in general, the CPI lays the foundations for new context-aware applications that will benefit from the availability of rich information from the hyper-map. For example, hyper-maps incorporating data on the spatial distribution of RF interference and sources of harvestable “clean” energy might significantly improve cognitive radio and energy harvesting schemes. Other applications are autonomous navigation of robots (including drones) in factories and/or hazardous scenarios and safe guidance in unknown and dangerous environments (e.g., during firefighters’ operations) or the provision of personalized health information to predict the personal exposure to potential risks (e.g., chemical or RF pollution).

In this paper, we have identified the main issues to realize the CPI. Most of them have still to be addressed or are only at an early stage. It is clear that there is the need for a solid and general theoretical framework, based on an expertise currently dispersed among different fields, like communication theory, wireless sensor networks, computer science, machine learning, antenna and propagation theory, distributed signal processing, localization, and cognitive neuroscience. Succeeding in this effort will make the network fully conscious of the surrounding environment, thus opening unexplored opportunities for ICT toward the entire mapping of the “Big Thing”.

In the perspective of the IoS in 6G networks, the proposed CPI architecture is expected to contribute to bridge the gap between such a visionary paradigm, and current state-of-the-art technology. In fact, the CPI intends to properly map the physical world into the virtual space, thanks to the adoption of the aforementioned networks of sensors, crowd-sensing processes and cognitive capabilities, thus becoming our digital sixth sense. In such an innovative scenario, it is also expected that augmented reality and virtual reality, greatly empowered by the CPI, will play a key role in enhancing the human experience of the real world [60].
